# Synthesis of freestanding HfO_2 _nanostructures

**DOI:** 10.1186/1556-276X-6-294

**Published:** 2011-04-05

**Authors:** Timothy Kidd, Aaron O'Shea, Kayla Boyle, Jeff Wallace, Laura Strauss

**Affiliations:** 1 Physics Department, University of Northern Iowa, Cedar Falls, IA 50614, USA; 2 Chemistry and Biochemistry Department, University of Northern Iowa, Cedar Falls, IA 50614, USA

## Abstract

Two new methods for synthesizing nanostructured HfO_2 _have been developed. The first method entails exposing HfTe_2 _powders to air. This simple process resulted in the formation of nanometer scale crystallites of HfO_2_. The second method involved a two-step heating process by which macroscopic, freestanding nanosheets of HfO_2 _were formed as a byproduct during the synthesis of HfTe_2_. These highly two-dimensional sheets had side lengths measuring up to several millimeters and were stable enough to be manipulated with tweezers and other instruments. The thickness of the sheets ranged from a few to a few hundred nanometers. The thinnest sheets appeared transparent when viewed in a scanning electron microscope. It was found that the presence of Mn enhanced the formation of HfO_2 _by exposure to ambient conditions and was necessary for the formation of the large scale nanosheets. These results present new routes to create freestanding nanostructured hafnium dioxide.

**PACS: **81.07.-b, 61.46.Hk, 68.37.Hk.

## Introduction

Owing to its high dielectric constant and lack of reactivity with silicon, hafnium dioxide has excellent characteristics for replacing SiO_2 _in nanometer scale applications such as gate oxides [1,2]. In addition to applications in electronics as thin films, there have been reports of interesting properties of HfO_2 _when synthesized in the form of nanocrystals or nanorods [3-5]. Inducing dimensional constraints by reducing the size of one or more dimensions has produced emergent phenomena in a range of materials such as graphene [6,7], single layer dichalcogenides [8], and other two-dimensional systems [9]. An example for the HfO_2 _system was that defect concentrations are easier to control when the HfO_2 _is formed as nanorods [4]. These defects can induce ferromagnetism, which has been far more difficult to reproduce in macroscopic HfO_2_.

With regards to nanostructure synthesis, the creation of two-dimensional freestanding nanostructures is of special interest. Most device applications entail the use of materials in the form of thin films. Determining the intrinsic properties of such films is difficult. Properties of the interfaces between the film and other components of the device can obscure the intrinsic properties of the film, and the interfacial effects only become larger as film thickness is decreased to nanometer scale dimensions. This issue has in part led to the development of synthesis techniques for creating various materials as freestanding, two-dimensional nanostructures [8-11].

In this work, we report two new methods for creating nanostructured HfO_2_. We have synthesized nano-scale crystallites of HfO_2 _as well as highly two-dimensional freestanding HfO_2 _nanosheets as a byproduct of the synthesis of HfTe_2_. The nano-scale crystallites were formed as a natural decomposition product from exposing HfTe_2 _to ambient conditions. The freestanding, two-dimensional oxide structures were induced to grow using a slightly modified growth process that normally yields HfTe_2 _in powder form. Both processes are extremely simple and represent new routes for synthesizing nanostructured HfO_2 _that could lead to new routes for inducing dimensional constraints in this material. Furthermore, as the HfO_2 _nanocrystallites are formed from the decomposition of powdered HfTe_2_, which is a layered material, it is expected that these structures are highly two-dimensional as well.

## Experimental methods

A mixture of HfTe_2 _and HfO_2 _was synthesized using standard techniques for growing transition metal dichalcogenides. Stoichiometric amounts of Hf and Te powders (Alfa Aesar, >99% purity) were added to a fused silica ampoule that was typically 8 cm long with a 1.1 cm inner diameter. The ampoules were then sealed under vacuum at a pressure of less than 0.1 mTorr. Samples were first heated to 125°C for 24 h to ensure that the ampoules would not burst from over-pressurization due to tellurium. The annealing temperature was then raised to 900°C and held at this temperature for several days. After the ampoules were opened, it was found that HfTe_2 _readily decomposed into HfO_2 _when exposed to ambient conditions. In most cases, it appeared that the original product was a powder consisting entirely of HfTe_2_, with HfO_2 _forming as a decomposition product after the ampoules were opened. Several attempts were also made to incorporate Mn or Cr dopants into the HfTe_2 _crystals. Doping levels up to a nominal 25% incorporation (i.e., Mn_0.25_HfTe_2_) were attempted for both elements. Powders of these elements (Alfa Aesar, >99.9% purity) would be mixed in various amounts with the original Hf and Te powders before the ampoules were sealed.

Sample products were measured using X-ray diffraction (XRD) with a Rigaku MiniFlex II. XRD measurements were performed on a silicon zero background sample holder for both powdered specimens and macroscopic HfO_2 _sheets. Powdered specimens were sifted through a -200 mesh (75 μm) sieve while larger sheets were laid flat upon the sample holder. X-ray analysis was performed using CrystalMaker™ software. The structural properties were measured using an Everhart-Thornley detector in a Tescan Vega II scanning electron microscope (SEM). Energy dispersive X-ray spectroscopy (EDS) was performed using a Bruker Quantax 400 system attached to the SEM. The images and EDS analysis shown here were performed using 20 kV electrons. Samples were fixed to aluminum posts for SEM measurements using double-sided carbon tape. Larger sheets were sufficiently stable for manipulation using tweezers and other instruments. Smaller powders were sifted onto the carbon tape for measurement.

## Results and discussion

The formation of HfO_2 _was actually an unintended consequence from attempts to grow pure and doped crystals of HfTe_2_. The actual products were a mixture of HfTe_2 _powders in the form of sub-millimeter crystals and products consisting of HfO_2_. It was also found that HfTe_2 _decomposed rather quickly into HfO_2 _upon exposure to air. The dopants, Mn or Cr, were never successfully incorporated into the main products, forming either impurity phases or ending up as a metallic residue on the walls of the ampoule. However, the inclusion of Mn did enhance the formation of HfO_2 _both during synthesis and after the samples were exposed to air.

**Figure 1:**
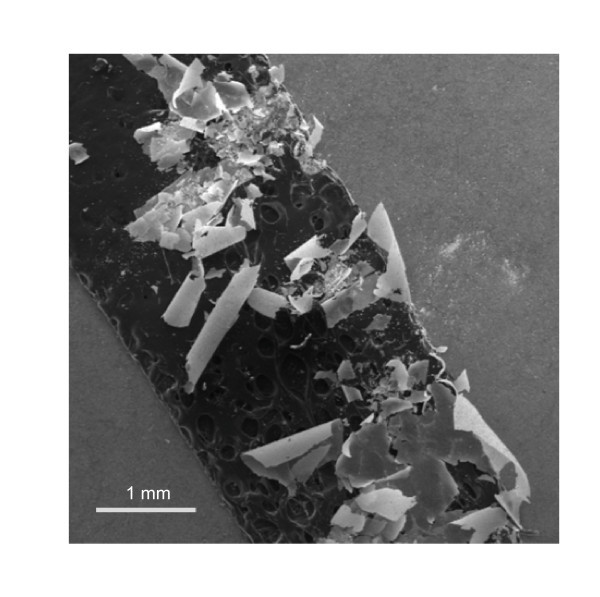
**SEM image of a collection of HfO_2 _nanosheets mounted on double sided carbon tape**. The sides of each sheet can be distinguished by their apparent brightness. During growth, the darker side was attached to the interior wall of the quartz ampoule.

In one set of samples, the heating cycle was performed twice without breaking vacuum. Of these samples, those containing Mn (nominal 25% doping) yielded a number of transparent sheets attached to the inner walls of the growth ampoule in addition to the usual HfTe_2 _powders. These sheets, larger examples of which can be seen in Figure 1, were barely detectable when the ampoules were first removed from the furnace. After some handling, but before the ampoules were cracked open, these sheets fell from the interior walls and landed on the HfTe_2 _powder contained within the ampoule. When this occurred, the mostly rectangular sheets rolled up so that the side exposed to the powder became the exterior. Their final curvature was much higher than would be expected from the 1.1 cm inner diameter of the silica ampoule.

It is not clear why the addition of Mn enhanced the formation of HfO_2_. Oxygen impurities in dichalcogenides have been reported in samples grown with manganese due to the manganese oxide which can readily form on powder Mn [12]. These samples also contained a larger than usual amount of MnTe impurity phase, thus reducing the overall amount of Te available for reaction and possibly inducing the Hf to scavenge small amounts of oxygen from the interior walls of the ampoules. After the ampoules were opened, the HfTe_2 _powders which contained Mn also converted to HfO_2 _more quickly, indicating the Mn might act as a catalyst for the oxidation reaction. This could also explain the enhanced formation of sheets within ampoules containing Mn. It is more likely that HfTe_2_, a relatively unstable compound, would be formed as an intermediate step before oxidation into HfO_2 _during the crystal growth rather than pure Hf scavenging oxygen its environment.

The HfO_2 _nanosheets were extremely thin considering their surface area, which ranged up to 25 mm^2^. These structures could be picked up with tweezers or otherwise manipulated for study by SEM, although some breakage and tearing occurred during handling. While somewhat brittle in their sensitivity to manipulation, the sheets were otherwise stable even after being studied for several months. The sheets showed signs of charging in the SEM, but not as much as might be expected from a wide gap insulator. As might be expected for a charging sample, edges of the sheet viewed at high magnification would tend to vibrate and wobble. This effect could be reduced by lowering the beam current and/or magnification. Bright and dark fringe patterns commonly seen on highly insulating materials like silica were not found, however. This indicates that the sheets behave more like semi-conducting materials than true insulators. This behavior is consistent with the presence of defects in the crystal lattice that would add carriers or reduce the band gap as has been seen in other examples of nanostructured HfO_2 _[4].

**Figure 2:**
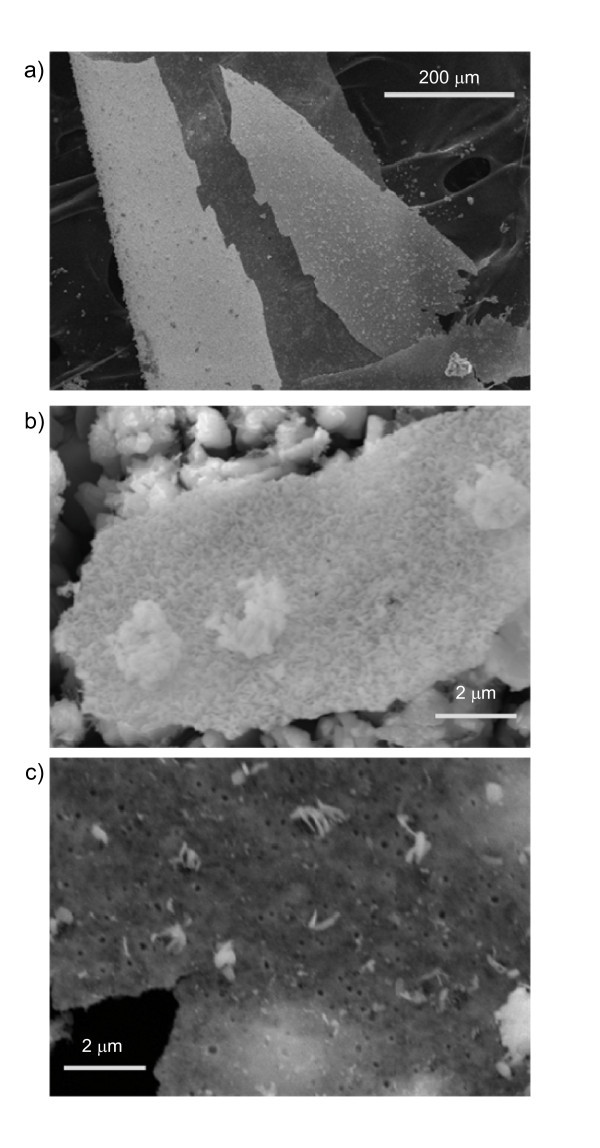
**SEM images comparing the bright and dark sides of HfO_2 _nanosheets**. **(a) **Wide view image of a curled sheet with a portion broken off. Bright and dark sides are both visible. **(b) **Close-up of the bright side. The surface has a lot of texture and contains micron scale clusters. Small dark circles can also be seen. **(c) **Close-up view of dark side. Surface is much smoother, although some particulate is attached. Small dark circles are again visible, measuring about 100 nm in diameter.

The differences between the two sides of these sheets can be more readily seen in Figure 2. The side that faced the interior of the growth ampoule has far more texture and contains a number of microscopic and sub-micron scale clusters. The large number of edges associated with these features makes this side appear brighter in the SEM. These clusters are well attached and likely formed during the growth process. The side that originally faced the ampoule walls appears darker in the SEM and is much smoother. There were far fewer particles attached to this side, and these particles sometimes seemed to shift position and their number increased as the samples were manipulated for various measurements. This indicates the particles on the smooth side appeared to be material that attached to the sheets after they were removed from the growth ampoule.

Another interesting feature common to both sides was the existence of small dark circles visible in Figure 2c. The size and spacing of these features was the same on both sides, indicating that they are likely pores in the structure. Measurements taken on the darker side, which were easier to focus on, showed that these features were all about 100 nm in diameter and surrounded by rings that were relatively bright compared to the rest of the surface. These dark spots were irregularly spaced but very consistent sizes, varying by less than 20%. While their origin is unclear, these features could arise from defect clusters induced by the high degree of anisotropy of the sheets. It is also possible that they could arise from crystal strain induced by a chemical reaction transforming hexagonal HfTe_2 _into monoclinic HfO_2_.

**Figure 3:**
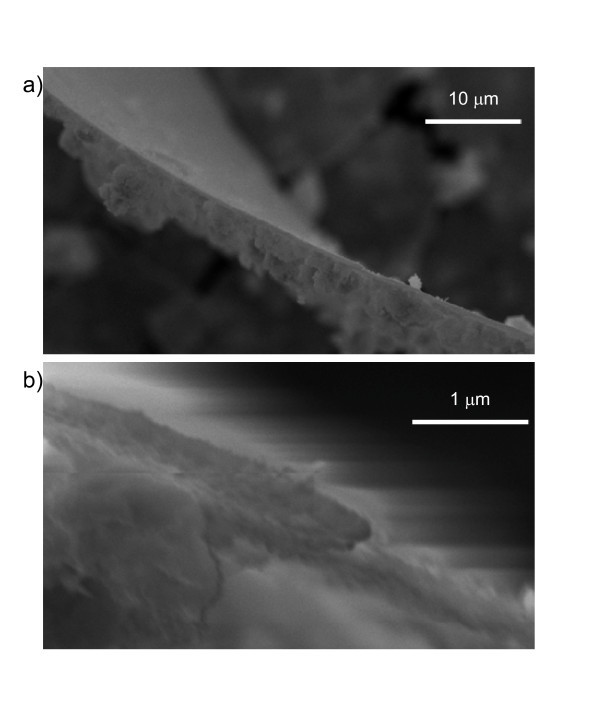
**SEM images of the edge of a HfO_2 _nanosheet**. (a) Wide view showing differences between smooth top side and cluster-filled bottom side. (b) Close-up of edge. Edge thickness is 200 nm.

The HfO_2 _sheets were so thin that, in the SEM, it was often possible to see through them and measure the pores of the carbon tape to which they were attached. Also, the larger clusters bound to the brighter side were often detectable as cloudy features (Figure 2c) seen when the darker side of the sheet faced the electron beam. It was possible to directly measure the thickness of a few of the larger sheets as they were bound to the carbon tape in a perpendicular fashion. The sheet shown in Figure 3 originally had side lengths that exceeded 1 mm, and after some fortuitous breakage became bound to the carbon tape by its edge. The differences between the bright (bottom) and dark (top) sides are readily apparent in the wide area view shown in Figure 3a, even though differences in relative intensity are muted when the sample is viewed at this angle. The dark side originally facing the quartz is almost featureless while the bright side is covered with clusters of various sizes. A higher magnification image of the edge is shown in Figure 3b. The thickness of the sheet itself, ignoring particulate or other clusters, was measured to be about 200 nm. Given that this was one of the thicker sheets, this implies that these HfO_2 _nanosheets are highly two-dimensional structures with dimensions similar to those used in thin film device applications.

**Figure 4:**
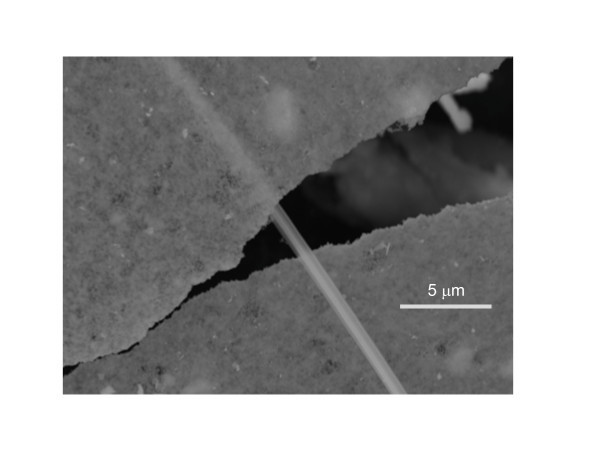
**SEM images of a bundled nanotube structure sandwiched between two HfO_2 _nanosheets**. The bundle can be easily seen through the transparent upper sheet.

It was apparent that different sheets had different thicknesses. Measurement of each was very difficult as mounting the sheets on edge was not a stable configuration and the sheets would often wobble or shift when high magnification measurements were attempted. However, one qualitative measure of sheet thickness that can be obtained in the SEM is their degree of transparency. In one area of the sample shown in Figure 4, a bundle composed of either nanotubes or nanorods was found trapped between two small HfO_2 _sheets. This was one of only a few bundles found in the sample, making it unclear whether this one-dimensional structure was an extremely rare growth product or if it was a contaminant from some bundled TaS_2 _nanotubes mounted on a different area of the sample stage in the SEM. Regardless of the bundle's origin, the image demonstrates just how transparent, and therefore thin, these sheets can be. The appearance of the bundle as seen through the upper sheet is smeared out, but not significantly dimmer compared to viewing it directly. This degree of transparency is similar to that of single-molecule thick materials [9].

The image of Figure 4 was taken using 20 kV electrons which have a mean free path of approximately 10 nm in most materials [13]. The secondary electrons measured in this image typically have energies less than 50 eV which have mean free paths on the order of 1 nm. To be imaged through the upper sheet, the electron beam had to pass through the sheet and create secondary electrons on the surface of the bundle. These secondary electrons would then need to pass through the sheet again to reach the detector. This could only occur if the sheet thickness was not more than a few nanometers, implying the entire structure was only several molecules thick. This represents an extremely large anisotropy, as this particular sheet was rectangular with sides measuring roughly 150 μm × 300 μm.

**Figure 5:**
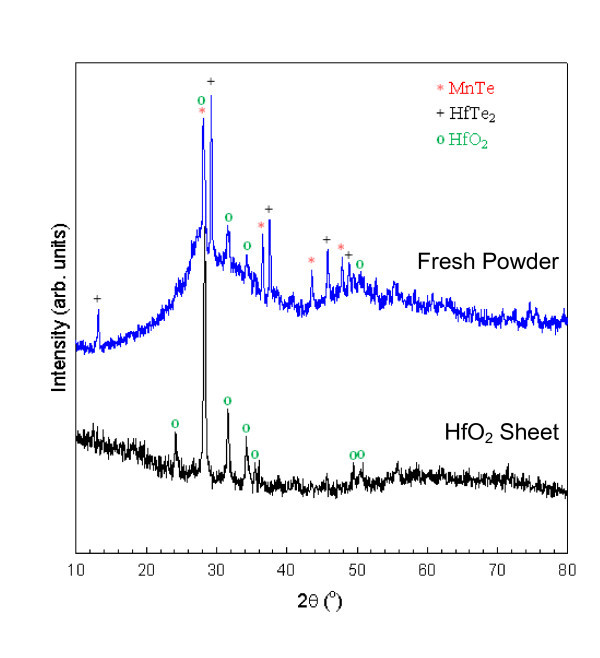
**XRD patterns from fresh powder and a relatively large HfO_2 _nanosheet**. Significant peaks related to the different phases are indicated by symbols.

A comparison of the XRD patterns taken from fresh powder and a relatively large HfO_2 _sheet are shown in Figure 5. The fresh powder was exposed to air for only a few hours while the sheet had been exposed to air for many days during sample handling and measurements. This powder and the sheets came from the same growth ampoule. The pattern from the fresh powder could be matched to peaks derived from HfTe_2 _[14], HfO_2 _[15], and MnTe [16] while the sheet pattern was essentially that of HfO_2_. The HfO_2 _sheet showed some enhancement of the  peak at 28.3° but not enough to definitively imply that the sheet was made up of a single, oriented crystal. The intensity of this peak was also enhanced in the powder sample, but this is likely due to an overlap with a MnTe peak located at 28.2°. The HfTe_2 _peaks showed significant (001) orientation from the intensity of the (002) peak at 13.4°, which should nominally be only 1.5% of the intensity of the main (011) peak found at 29.3°. This orientation is common for layered dichalcogenides in powder form as they are typically made up of small, thin platelets that are difficult to force into a random configuration.

**Figure 6:**
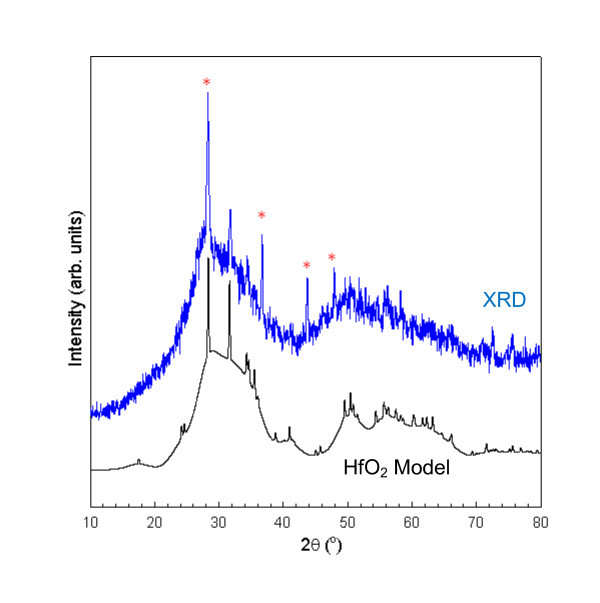
**Model and measured XRD pattern for aged powder sample**. The model is composed of a mixture of "macroscopic" (>50 nm) and nanometer scale HfO_2 _particles. The marked peaks indicate MnTe impurities not accounted for in the model.

Another interesting feature of the powder XRD pattern is the appearance of the background in the spectra. It appears as if there are a large number of extremely broad states that underlie the sharp Bragg peaks in the spectrum of the powder sample. To better understand this phenomenon, the powder was left exposed to air for some time, which resulted in all traces of the HfTe_2 _disappearing from the sample. The XRD pattern of this aged powder is shown in Figure 6. The only peaks remaining, aside from the anomalous background, can be attributed to HfO_2 _and the MnTe impurity phase. The model is actually a simple mixture of a simulated XRD pattern composed of 5% "macroscopic" and 95% nanometer scale HfO_2 _particles with a mean diameter of 2 nm. In this case, "macroscopic" means only that the material is sufficiently large (>50 nm) so that the peaks are not overly broadened as compared to the sharp features in the data. The model is quite simple, ignoring all broadening effects aside from particle size. The features are essentially too broad for other parameters, such as strain, to be of much significance. The model does not include any attempts to actually fit the data by introducing background effects, orientation, or any other parameters. Instead, it is meant to show that the major features of the data can be well reproduced by assuming the powder a mixture composed mainly of randomly oriented HfO_2 _particles with nanometer scale sizes along with some larger HfO_2 _particles. The only features that are not accounted for in the model are those associated with MnTe impurities. The impurities are the source of sharp peaks near 36.7°, 43.7°, and 48° as well as the enhancement of the HfO_2 _peak near 28.3°. The success of this model supports the SEM findings that the freestanding HfO_2 _sheets are extremely anisotropic materials with nanometer scale thicknesses.

## Conclusions

Freestanding two-dimensional nanosheets of HfO_2 _and nanometer scale HfO_2 _crystallites were synthesized as byproducts of the attempted growth of pure and doped HfTe_2_. The oxide growth was enhanced by the presence of Mn in the growth ampoule in both cases. It appears as if the HfO_2 _sheets were formed during the growth process while the nanometer scale crystallites formed after the ampoules were cracked open and the resulting HfTe_2 _powders were exposed to air. While it is not clear exactly what form the nanometer scale HfO_2 _crystallites have, it would not be surprising if they were two-dimensional as well given that their precursor, HfTe_2_, is itself a highly two-dimensional layered material. Given that it is possible to exfoliate dichalcogenides to create single molecular layers [8], this synthesis route could be able to yield two-dimensional nanostructures in any case.

The HfO_2 _sheets were extremely two-dimensional with thicknesses ranging from a few nanometers to no more than a few hundred nanometers. In addition to being extremely thin for their size, they also contained a large number of defects in the form of sub-micron scale holes. It is not clear what effect these structures have, but they could relate to other vacancy type defects that have been shown to influence magnetic behaviors in nanostructured HfO_2_. These results represent a new route for synthesizing nanostructured HfO_2 _and the first reported example of freestanding two-dimensional HfO_2 _nanostructures.

## Abbreviations

EDS: energy dispersive X-ray spectroscopy; SEM: scanning electron microscope; XRD: X-ray diffraction.

## Competing interests

The authors declare that they have no competing interests.

## Authors' contributions

AO and JW performed the microscopy and chemical analysis. KB and LS carried out the X-ray diffraction measurements and synthesis. TK wrote the manuscript, directed measurements, and performed analysis of the structural and chemical properties. All authors read and approved the final manuscript.
